# Enhanced Photoluminescence of Hydrogenated Amorphous Silicon Carbide Thin Films by Means of a Fast Thermal Annealing Process

**DOI:** 10.3390/ma13112643

**Published:** 2020-06-10

**Authors:** Israel Vivaldo, Roberto C. Ambrosio, Roberto López, Javier Flores-Méndez, Luis A. Sánchez-Gaspariano, Mario Moreno, Filiberto Candia

**Affiliations:** 1 Electronics Department, Benemérita Universidad Autónoma de Puebla, Puebla 72590, Mexico; javier.floresme@correo.buap.mx (J.F.-M.); luis.sanchezgas@correo.buap.mx (L.A.S.-G.); filiberto.candia@correo.buap.mx (F.C.); 2 Mechatronics Department, Tecnológico de Estudios Superiores de Jocotitlán, Carretera Toluca-Atlacomulco km 44.8, Ejido de San Juan y San Agustin, Jocotitlán 50700, Mexico; roberto.lopez@tesjo.edu.mx; 3 Tecnológico Nacional de México/I.T. Puebla-División de Estudios de Posgrado e Investigación, Av. Tecnológico No. 420, Maravillas, Puebla 72220, Mexico; 4 Electronics Department, Instituto Nacional de Astrofísica, Óptica y Electrónica, Puebla 72000, Mexico; mmoreno@inaoep.mx

**Keywords:** photoluminescence, amorphous silicon carbide, fast thermal annealing process, plasma enhancement chemical vapor deposition

## Abstract

In this paper, the photoluminescence (PL) of hydrogenated amorphous silicon carbide (a-Si_1−x_C_x_:H) thin films obtained by Plasma Enhancement Chemical Vapor Deposition (PECVD) is reported. Strong PL is obtained after a fast annealing process for 60 s at temperatures of 200, 400, 600, and 800 °C. The thin films are characterized using Fourier Transform Infrared spectroscopy (FTIR), PL spectroscopy, and Energy-Dispersive X-ray Spectroscopy (EDS). According to the results of the structural characterization, it is deduced that a structural rearrangement of the amorphous matrix is carried out during the fast annealing process, which results in different degrees of oxidation on the a-Si_1−x_C_x_:H films. The PL peak position shifts towards higher energies as the temperature increases. The sample deposited with a silane/methane flux ratio of 37.5 at an Radio Frequency (RF) power of 6 W experiences an increase in PL intensity of more than nine times, with a displacement in the peak position from 2.5 eV to 2.87 eV, at 800 °C. From the PL analysis, we observe two emission bands: one centered in the near infrared and other in the visible range (with a blue peak). This study opens the possibility to use such thin films in the development of optoelectronics devices, with potential for application in solar cells.

## 1. Introduction

The amorphous and crystalline phases of silicon carbide (SiC) materials have been extensively investigated in the field of semiconductor devices, specifically regarding the development of optoelectronic devices such as solar cells and light-emitting diodes. For example, the hydrogenated amorphous phase of silicon carbide (a-SiC:H) has been demonstrated to be a good candidate for the development of large-area optoelectronic devices due to its light emission and absorption properties. Different methods for obtaining a-SiC:H thin films have been used, such as electron cyclotron resonance (ECR), magnetron sputtering, and Plasma Enhanced Chemical Vapor Deposition (PECVD) [1,2,3]; the latter is one of the most extensively used methods, due to the low temperature of deposition and the possibility of integration with silicon technology.

Strong photoluminescence (PL) is an essential property in materials used for photon conversion phenomena such as down-conversion, up-conversion, and luminescent down-shifting. These important processes have been used to improve the spectral response, external/internal quantum efficiency, and efficiency of solar cells [4,5,6]. For this purpose, several authors have reported photoluminescence in a-SiC:H. Vasin et al. [2] reported the strong PL of a-SiC:H films deposited by sputtering with an annealing process at 450 °C for 30 min in dry oxygen, with values in the range of 1.91–3.26 eV with a peak at 2.66 eV. Li et al. [7] obtained luminescence values from 1.75 to 3.1 eV with a peak at 2.48 eV of a-SiC:H using a PECVD system with high power densities (289–520 mW/cm^2^). Due to long deposition times and high power densities producing thick films, where uniformity decreases as thickness increases, the application of such technologies could be complicated. On the other hand, Wen et al. [8] performed thermal annealing processes on a sample of a-SiC:H at different temperatures, obtaining strong PL at temperatures above 1000 °C, with peaks at 2.1, 2.58, and 2.88 eV on PL.

Therefore, the research works on a-SiC:H have reported different bands of light emission, some of which have associated the PL to quantum confinement effects (if there are silicon nanocrystals in the amorphous matrix [1,8]) and others to radiative recombination in the band gap [7,9,10]; however, the majority have related the intensity and position of PL to the carbon content [2,11,12]. Nevertheless, there are few studies in which thermal annealing has been performed to study its effect on light emission.

In this work, thin films of a-Si_1−x_C_x_:H are obtained by the reaction of a mixture of methane (CH_4_) and silane (SiH_4_) gases in a Radio Frequency (RF) PECVD reactor, followed by a fast annealing process at different temperatures in a conventional quartz furnace for one minute in an inert N_2_ atmosphere to study its effect on the PL property. The thin films are characterized using Fourier Transform Infrared spectroscopy (FTIR), PL, and Energy-Dispersive X-ray Spectroscopy (EDS). The annealing temperature was found to have a great influence on the PL intensity and the position of the light emission band. A more than nine-fold improvement in the PL intensity with the annealing process at 800 °C for 60 s was obtained.

## 2. Materials and Methods

Hydrogenated amorphous silicon carbide films were deposited on n-type < 100 > c-Si wafers in a parallel electrode High Vacuum PECVD reactor working at a RF of 13.56 MHz from MVSystem. The precursor gases for the gas mixture were CH_4_ and SiH_4_ (at 10% in H_2_) at a substrate temperature of 150 °C and pressure of 0.7 Torr, with a deposition time of 20 min while the RF power varied from 6–15 W. The CH_4_/SiH_4_ gas ratio was varied, with the CH_4_ gas flow constant at 30 sccm while the SiH_4_ gas flow rate varied in the range of 8–4 sccm. The thickness of the films was evaluated with an optical profiler (model KLA P–7 Stylus Profiler). The purpose of varying the CH_4_/SiH_4_ gas ratio was to produce a-Si_1−x_C_x_:H films with different carbon (C) content, to study its influence on the photoluminescent characteristics of the films. Table 1 shows the conditions of deposition for the a-Si_1−x_C_x_:H films.

The as-deposited films were divided into four parts, to apply fast annealing at different temperatures: 200, 400, 600, and 800 °C. The fast annealing was carried out in a conventional hot-wall reactor in a nitrogen environment for 60 s; the cooling process was carried out by leaving the samples at room temperature.

The PL intensity at room temperature of the a-Si_1−x_C_x_:H films was measured using a Horiba Jobin Yvon Fluoro-Max3 spectrofluorometer. The films were excited using a 330 nm light beam with 75.1 µW power; employing an optical filter, the emission signal was collected from 400 to 950 nm with a resolution of 1 nm. Fourier Transform Infrared (FTIR) spectroscopy was used to characterize the absorbances of the a-Si_1−x_C_x_:H films in the range of 400–4000 cm^−1^ with a resolution of 2 cm^−1^. The infrared absorption spectra were obtained with a Bruker Vector 22 spectrometer. The PL intensity depends on the sample thickness; therefore, we carried out normalization of the PL and FTIR spectra to the thickness of each film. This normalization was also useful in studying the evolution of each bond with the temperature and the influence of the bonds to the PL intensity. To quantify the Si and C solid content of the films, EDS was employed using a Bruker Energy-Dispersive X-Ray Spectrometer QUANTAX 200.

## 3. Results and Analysis

During the thin film deposition, SiH_4_, CH_4_, and H_2_ gases are first decomposed into SiH_n_, Si, CH_n_, C, and H by the RF glow discharge. Then, the plasma reacts to generate Si–C, Si–CH_n_, and Si–H_n_ bonds, among others. Due to the presence of CH_n_, it is difficult to decompose into C atoms, compared to SiH_n_; although both SiH_n_ and CH_n_ are decomposed further by increasing the RF deposition power, more SiH_n_ than CH_n_ is decomposed under high RF deposition power. Therefore, the amorphous matrix obtained contains more silicon than carbon. To elucidate the effect of fast annealing on structural and light-emitting properties, the results are presented first without annealing and then compared with the annealing process.

**Figure 1:**
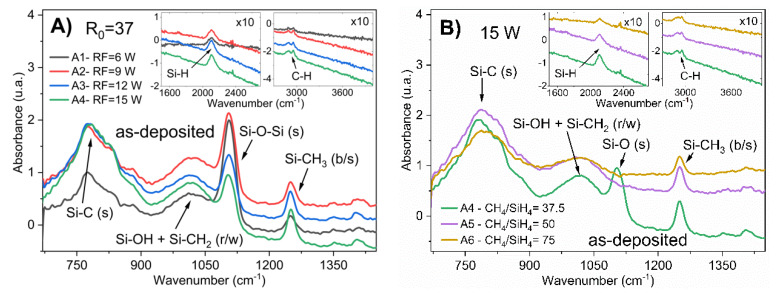
FTIR spectra of as-deposited a-Si_1−x_C_x_:H films: (**A**) with different RF power (6–15 W) at a constant ratio of CH_4_/SiH_4_ (R_0_ = 37.5); and (**B**) corresponding to films deposited with different CH_4_/SiH_4_ ratios (R_0_ from 37.5 to 75) with a constant RF power of 15 W.

FTIR spectra revealed several bonding components in the as-deposited a-Si_1−x_C_x_:H material. Figure 1A corresponds to a-Si_1−x_C_x_:H films deposited with different RF powers (6–15 W), keeping the CH_4_/SiH_4_ ratio constant; in contrast, Figure 1B corresponds to a-Si_1−x_C_x_:H films deposited with different CH_4_/SiH_4_ ratios (R_0_ from 37.5 to 75) with a constant RF power (15 W).

Considering the FTIR spectra of the as-deposited films, four main absorption bands can be observed: at 780 cm^−1^, the Si–C stretching mode is located [1,13,14]. The absorption band centered at 1015 cm^−1^ is related to the rocking/wagging vibration modes of CH_2_ radicals bonded to a silicon atom (Si–CH_2_) [15]; however, this band absorption can also be related to OH radicals bonded to silicon (Si–OH) [16]. The strong and narrow peak at 1104 cm^−1^ is associated with Si–O–Si stretching vibration. As the CH_3_ symmetric (“umbrella”) deformation is strongly dependent on the electronegativity of the adjacent atom, the Si–CH_3_ vibration is located at 1250 cm^−1^ [17]. Weak absorption was observed at ~1400 cm^−1,^ which is attributed to the C–H bending. Additionally, some absorption bands in the ranges of 2200–2000 cm^−1^ and 3050–2850 cm^−1^ were present, due to the Si–H and C–H stretching modes, respectively [13,15]. 

The FTIR measurements suggest the absence of a noticeable concentration of graphitic-like bonding configurations in the a-Si_1−x_C_x_:H, as no peaks were observed at 3000 cm^−1^. It is important to remark that the FTIR measurement performed in an ambient nitrogen atmosphere could eliminate the false signals of triple bonds and cumulated double bonds (X≡Y and X=Y=Z) in the range of 2300–1900 cm^−1^ [16].

From the FTIR spectra, we can also observe some oxygen bonds, even when the a-Si_1−x_C_x_:H films were deposited with a vacuum system and without any oxygen (O) flow; therefore, there was some incorporation of O into the films, which could be attributed to some contaminants such as atmospheric oxygen (CO_2_, H_2_O, N_2_), due to vacuum leaks, impurities in the gases, pump oil backstreaming (hydrocarbons), or from the absorption of reactor surfaces (H_2_O) [18]; moreover, the PECVD deposition parameters seem to have influenced the percentage of O incorporation. Therefore, the films could be considered to be an alloy composed of Si, C, and O; that is, an amorphous silicon hydrogenated oxycarbide [14,19].

The FTIR characterization provides information about the chemical bonding types which are present in the films, but is not very suitable for determining the content of each element. To clarify the compositional content of the films, EDS was employed. 

Table 2 shows the atomic compositions obtained through the EDS characterization of the different a-Si_1−x_C_x_:H films. From the results, it can be observed that as the RF power increased, the Si content decreased; from 59. 28% at low RF power (6 W) to 50.02% at high RF power (15 W). At low RF power (6 W), the C content was 22.81%, which increased with the RF power; at high RF power (15 W), the C content was 36.61%.

According to the results of EDS, the carbon concentration was low for low RF powers (6 W). In addition, from the FTIR spectra shown in Figure 1A, it is possible to observe that the use of low RF power (6 W) limited the formation of silicon–carbon bonds to obtain silicon carbide; thus, sample A1 was mainly composed of Si–O bonds and CH_2_ and CH_3_ radicals bonded to silicon. Samples deposited with a greater RF power than 6 W had a higher content of silicon–carbon bonds. 

For low silane rates (R_0_ = 50 and 75), the content of oxygen decreased. The FTIR spectra also presented a decrease in the silicon–oxygen bonds. In this way, the main absorption band was focused on silicon–carbon bonds. The A5 and A6 films were composed of silicon carbide, with CH_2_ and CH_3_ radicals bonded to silicon and a low concentration of Si–O bonds, as shown in Figure 1B.

**Figure 2:**
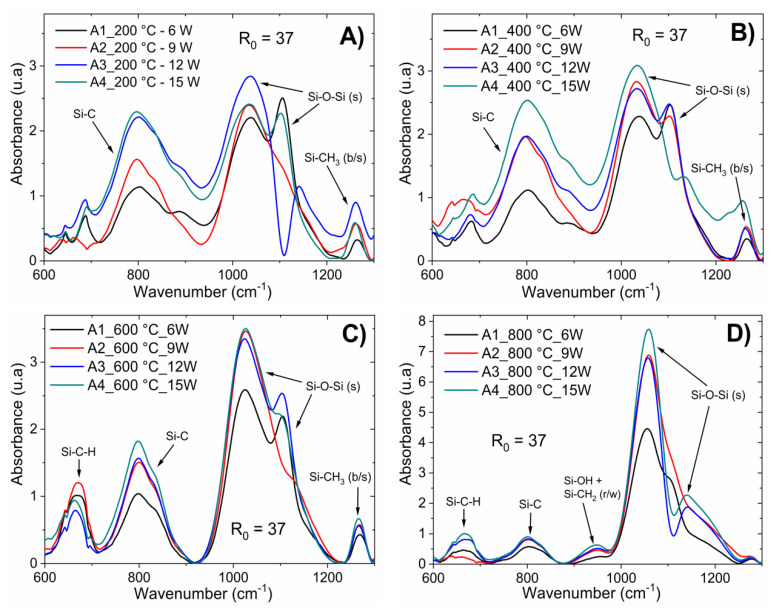
IR spectra for the a-Si_1−x_C_x_:H films deposited after fast annealing process with different RF power (from 6–15 W) while keeping the CH_4_/SiH_4_ ratio constant; upon fast annealing process at 200 (**A**), 400 (**B**), 600 (**C**), and 800 °C (**D**).

**Figure 3:**
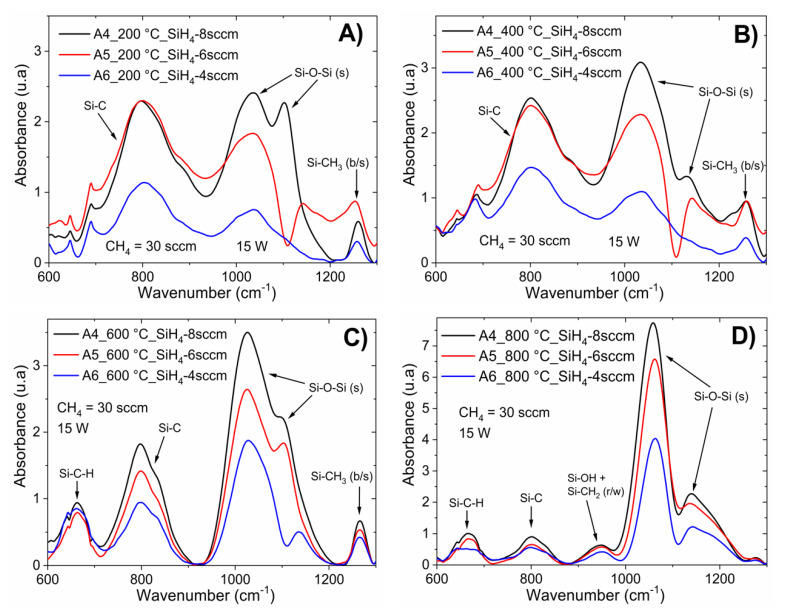
IR spectra for the a-Si_1−x_C_x_:H films deposited after fast annealing process with different R_0_ ratios (from 37.5–75) and constant RF power at 15 W; upon fast annealing process at 200 (**A**), 400 (**B**), 600 (**C**), and 800 °C (**D**).

Chemical bonding was also observed to be dependent on the post-deposition thermal processing. IR spectra analysis for the six samples of a-Si_1−x_C_x_:H with annealing at 200, 400, 600, and 800 °C are shown in Figure 2 and Figure 3. Figure 2 corresponds to a-Si_1−x_C_x_:H films deposited with different RF powers (6–15 W) keeping the CH_4_/SiH_4_ ratio constant; while Figure 3 corresponds to a-Si_1−x_C_x_:H films deposited with different CH_4_/SiH_4_ ratios (R_0_ from 37.5 to 75) with constant RF power (15 W).

The thin film samples were rapidly heated homogeneously. Thus, consequently, enough energy was gained for structural rearrangement. The main changes observed after annealing occurred in the Si–O–Si bonds in their different phases; as the temperature increased, the intensity of the absorption peak associated with this type of bond increased. This was correlated with the deposition parameters, as shown in Figure 2 and Figure 3. The increase in Si–O–Si bonds indicates that as the temperature increased during the annealing process, the degree of oxidation of the sample increased, which was observed for the temperatures of 600 and 800 °C. After the rapid thermal annealing, the Si–OH and Si–CH_2_ bonds disappeared while for temperatures above 600 °C, a new absorption band centered at 663 cm^−1^ appeared, which is associated with Si–C–H bonds. The absorption band related to Si–C bonding shifted to low frequencies and was centered at 808 cm^−1^, as shown in labels (C) and (D) in Figure 2 and Figure 3. This structural rearrangement modified the light emission bands, as corroborated by the PL measurements.

**Figure 4:**
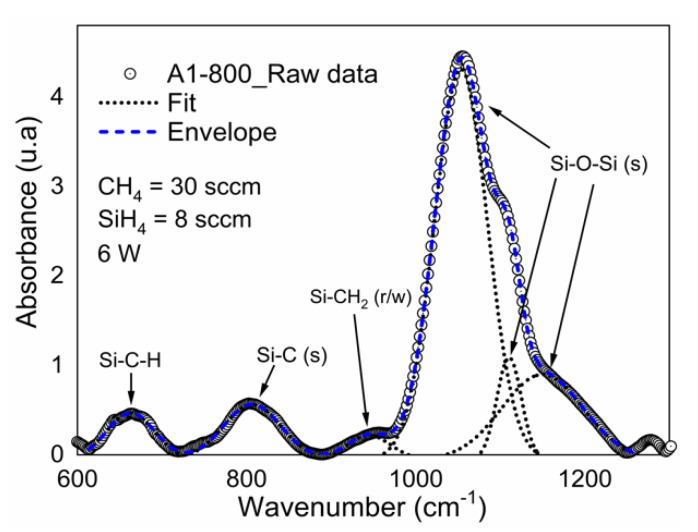
Deconvolution of A1 sample (6 W and R_0_ = 37.5) upon fast annealing process at 800 °C.

To confirm the degree of oxidation, the deconvolution of the A1 sample (6 W and R_0_ = 37.5) with thermal annealing at 800 °C is shown in Figure 4. The Levenberg–Marquardt algorithm was used for the deconvolution with a confidence of 95%.

Deconvolution of the main absorption band (Si–O–Si) shows three peaks centered at 1053 cm^−1^, 1110 cm^−1^, and 1149 cm^−1^. The asymmetric stretching vibrations in which O atom motion occurs in the Si–O–Si plane parallel to a line joining the two silicon atoms are suggested to be responsible for the strongest absorption near 1053 cm^−1^ [13]. The presence of the three Si–O asymmetric stretching bands suggests the existence of three amorphous SiO_x_ phases, as correlated with changes in the Si–O–Si bonding angle. The peak at 1053 cm^−1^ is attributed to a smaller Si–O–Si bond angle, considering the bond angle for stoichiometric silicon dioxide (144°), for which the peak is at 1080 cm^−1^.

In this way, the main objective of this work is to enhance the light emission (PL) produced by a-Si_1−x_C_x_:H films when they are irradiated with ultraviolet (UV) light. The mechanisms that have been proposed to explain the photoluminescence in a-Si_1−x_C_x_:H are diverse, some of the main explanations are excitonic emission, quantum confinement of carriers in silicon nanocrystals inside the amorphous matrix, and emission due to localized defects.

**Figure 5:**
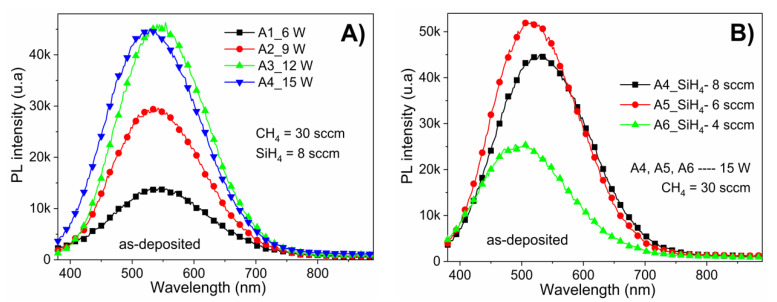
PL spectra of as-deposited a-Si_1−x_C_x_:H films: (**A**) with different RF power (6–15 W) keeping the CH_4_/SiH_4_ ratio (R_0_ = 37.5) constant; and (**B**) films deposited with different CH_4_/SiH_4_ ratios (R_0_ from 37.5 to 75) at constant RF power (15 W).

Figure 5A shows the PL spectra of as-deposited a-Si_1−x_C_x_:H films with different RF power (6–15 W) while keeping the CH_4_/SiH_4_ ratio (R_0_ = 37.5) constant. From these spectra, it can be observed that the RF power had a strong effect on the PL intensity, with a clear increase of PL intensity observed as the RF power increases. Furthermore, the samples present luminescence throughout practically the entire visible range (i.e., 400–700 nm) with a peak intensity at 542 nm (2.28 eV). Notice that despite the PL intensity being affected by the deposition RF power, its Gaussian shape is not affected. The increase from 12 to 15 W in deposition power did not induce an increase in the photoluminescence intensity; however, there was a shift in the emission band (as the peak is located at 2.34 eV). These results are consistent in agreement with some previous studies [20,21].

The PL results showed significant changes in the intensity as a function of silane rate; however, no trend could be identified. In Figure 5, the PL peak position shifts from 530 nm (at 2.33 eV) to 490 nm (at 2.53 eV) when the CH_4_/SiH_4_ flow rate ratio increases from 37.5 to 75. This shift could be due to changes in the amorphous matrix, as shown in Figure 1B, causing stress in the localized states resulting in light emission.

**Figure 6:**
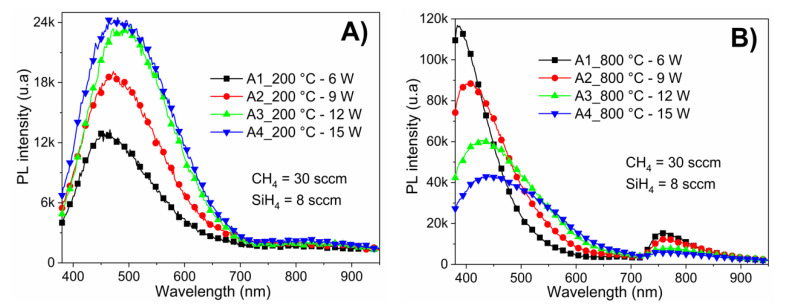
PL spectra of a-Si_1−x_C_x_:H films deposited with different RF power (6–15 W) while keeping the CH_4_/SiH_4_ ratio (R_0_ = 37.5) constant: (**A**) films annealed at 200 °C; and (**B**) films annealed at 800 °C.

PL spectra after annealing treatment of a-Si_1−x_C_x_:H films deposited with different RF powers and constant ratio R_0_ =37.5 are shown in Figure 6A,B, annealed at 200 and 800 °C, respectively. Samples annealed at low temperature (200 °C) had a lower luminescence intensity than samples annealed at high temperature (800 °C); furthermore, the maximum emission peaks shifted towards shorter wavelengths (higher energies) as a function of the annealing temperature.

In sample A1, the PL peak position of the as-deposited film was located at 544 nm, which shifted to 461 nm when annealing at 200 °C, and to 385 nm when annealing at 800 °C.

Notice that the samples annealed at high temperature (800 °C), Figure 6B have two emission bands; sample A2 (R_0_ = 37.5 and RF power = 9 W) has an intense emission band over almost the entire visible range (380–602 nm) with a maximum peak at 405 nm, as well as another low-intensity emission band in the near infrared (728–847 nm). Samples annealed at 200 °C only have one PL emission band covering almost the entire visible range (380–706 nm), as shown in Figure 6A. In fact, samples with annealing at 600 and 800 C show two emission bands; while those annealed at 200 and 400 only have one band. The emission band in the near infrared have been reported in silicon oxides [22,23]; this suggests that the oxidation degree increases with the annealing temperature. In the supplementary material (Figures from S1–S10) section, a qualitative comparison of normalized photoluminescence for different conditions of deposition is shown.

**Figure 7:**
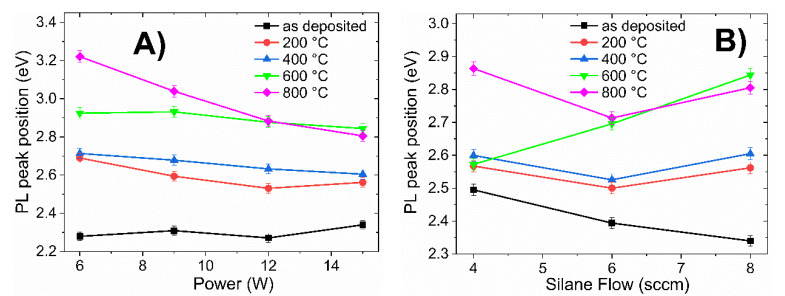
PL peak position of a-Si_1−x_C_x_:H films deposited: (**A**) with different RF power (6–15 W) keeping the ratio R_0_ constant (37.5); and (**B**) with different R_0_ (37.5–75) with constant RF power (15 W).

To summarize the PL results, Figure 7 presents the maximum PL peak position of all films as a function of RF power, R_0_ ratio, and annealing temperature. Figure 7A corresponds to films with different RF powers (6–15 W) at R_0_ = 37.5; Figure 7B corresponds to those films deposited with a fixed RF power (15 W) and differing R_0_ (37.5–75).

From Figure 7A the PL peak position in as-deposited films is practically kept constant as a function of power (black line in Figure 7A); however, after fast annealing, the PL peak position strongly depends on the annealing temperature. All samples deposited with R_0_ =37.5 presented a shifting in the peak and light emission band towards the blue as the annealing temperature increased, regardless of the deposition power. This correlated with the FTIR results and corresponded to an increase in the degree of oxidation. The effect was noticeable for the film deposited with a low RF power (A1; 6 W), with PL peak located at 2.28, 2.69, 2.71, 2.92, and 3.22 eV for the sample without annealing and annealing temperatures at 200, 400, 600, and 800 °C, respectively. The PL peak position experienced a 0.94 eV (159 nm, from green to violet) strong shift towards higher energies. 

According to X-ray Diffraction (XRD) and High-Resolution Transmission Electron Microscopy (HRTEM) measurements, the structural matrix was amorphous in the deposited samples; there was no evidence of the presence of silicon nanocrystals before or after the annealing process (see supplementary material, Figure S11). Therefore, the displacement PL peak position cannot be associated with carrier quantum confinement. Thus, the light emission of the films obtained in this work was associated with recombination processes in localized states and the change in the PL peak position was due to changes in the bond angles of the luminescence centers. For example, in sample A1 (6 W and R_0_ = 37.5), the shift of the PL peak position was accompanied by a strong increase in the light emission intensity, which suggests that the increase of light emission was due to an increase in the recombination centers in the amorphous silicon oxycarbide; the alloys formed during the rapid annealing process.

Shifting of the PL peak position was also observed for the samples deposited with different R_0_ ratio, as shown in Figure 7B. The displacement of PL peak position was small for the sample deposited with high ratio R_0_ (75) at temperatures below 600 °C (i.e., 0.1 eV with respect to the sample without annealing); however, when the temperature rose to 800 °C, an abrupt shift in PL peak position can be observed (from 2.5 eV to 2.87 eV).

According to the EDS results, as the silane flow decreases, the oxygen content decreases; thus, low silane flow limits the incorporation of oxygen. Figure 3C shows the FTIR spectra for the samples deposited with different silane flows annealed at 600 °C, from which it can be observed that the oxygen content had a strong influence on bond rearrangement during annealing: as the oxygen content decreased, the intensity of the asymmetric Si–O–Si stretching band decreased and shifted toward higher wavenumbers. The decrease intensity and shifting of this absorption band caused a strong shift in the PL peak position, as shown in Figure 7B.

**Figure 8:**
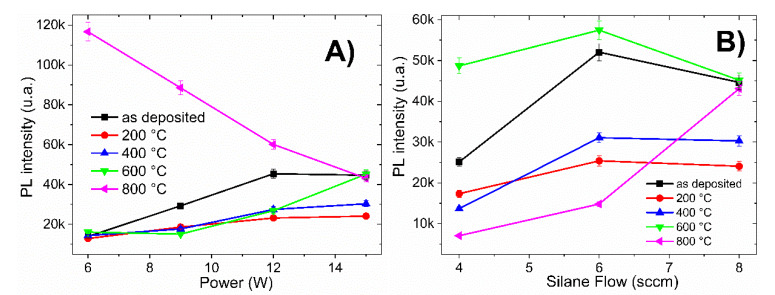
PL intensity of a-Si_1−x_C_x_:H films deposited: (**A**) with different RF power (6–15 W) maintaining constant R_0_ ratio (37.5); and (**B**) with different R_0_ ratios (37.5–75) and fixed RF power (15 W).

Another important aspect to consider is the change in PL intensity with respect to the annealing temperature. Figure 8 presents the PL intensity as a function of RF power, CH_4_/SiH_4_ ratio, and annealing temperature. Figure 8A corresponds to films with different RF powers (6–15 W) keeping the CH_4_/SiH_4_ ratio (R_0_ = 37.5) constant; while Figure 8B corresponds to films deposited with different CH_4_/SiH_4_ ratios (R_0_ = 37.5–75) at constant RF power (15 W).

The samples annealed at high temperatures (800 °C) showed a strong improvement in PL intensity when the gas flow ratio and the RF power deposition were low (R_0_ = 37.5; 6 W, 9 W, and 12 W), as shown in Figure 8A. Meanwhile, for samples deposited with low silane flow (4 sccm and 6 sccm in Figure 8B, the maximum PL intensities were obtained with annealing at 600 °C.

The PL peak position of all films with thermal treatment at temperatures below 400 °C (Figure 7) shifted towards blue, depending in the degree of oxidation and the carbon, oxygen, or silicon content; however, the intensity of light emission decreased and there was only one emission band.

Sample A1 (R_0_ = 37.5 and 6 W RF power) showed an increase in PL intensity of more than nine times with annealing temperature at 800 °C, in contrast to the sample without annealing, as can be observed in Figure 8A. From the EDS results and Figure 8B, when the sample has a high carbon content and a low oxygen content, an improvement in PL intensity is obtained at 600 °C.

Many structural defects in silicon-based materials, such as Si-related oxygen deficiency centers (Si-ODCs; e.g., O3≡Si…Si≡O3), non-bridging oxygen hole centers (NBOHCs) whose structure is identified by its paramagnetic properties and is denoted by ≡Si-O• (where (≡) stands for bonds with three oxygens and (•) indicates an unpaired electron), Si-related oxygen vacancies (Si-NOVs), and C-related oxygen vacancies (C-NOVs), are known to be luminescent in the UV/visible range. For example, NBOHCs yield red (~2 eV) emission, while Si-ODCs and Si-NOVs or C-NOVs result in green and blue emission, respectively, in the ranges of 2–2.4 eV and ~2.8 eV [7,10,24,25]. Therefore, the dynamic behavior of photoluminescence observed in the deposited films can be inferred to have originated from defect states.

## 4. Conclusions

A study of the PL properties of hydrogenated amorphous silicon carbide films after applying a fast annealing process at different temperatures was carried out. According to the EDS analysis, the RF power during the deposition process delimits the incorporation of oxygen in the samples and the fast annealing process formed an alloy composed of silicon, carbon, and oxygen, resulting in an amorphous silicon oxycarbide.

The peak position and intensity of PL can be modified as a function of temperature, which is an important result because the displacement in the PL peak position has only been reported previously as a function of the carbon content. Samples deposited with a CH_4_/SiH_4_ gas flow ratio of 37.5 show an improvement in luminescence with annealing at 800 °C.

One of the most important contributions in this work is obtaining strong PL without a long annealing process at high temperature; therefore, these samples can easily be incorporated into complementary metal-oxide-semiconductor (CMOS) processes which include light-emitting devices. If strong PL is not required, the films without annealing process have good emission properties and allow the manufacture of devices at low temperatures in flexible substrates.

The strong PL obtained could be incorporated into silicon-based solar cell technology, with the advantage of being able to use this material for the down-conversion or down-shifting of high energy incident photons.

As silicon-based amorphous materials have a wide variety of luminescent defects, further work is required to elucidate this issue and to determine the mechanisms that produce photoluminescence; this would be part of our future work.

## Figures and Tables

**Table 1:** Deposition parameters of a-Si_1−x_C_x_:H films deposited by RF PECVD at 150 °C.

Sample.	RF Power (W)	CH_4_ (100%) (sccm)	SiH_4_ (10% in H_2_) (sccm)	CH_4_/SiH_4_	Thickness (nm)
A1	6	30	8	37.5	140 ± 8
A2	9	30	8	37.5	186 ± 5
A3	12	30	8	37.5	208 ± 9
A4	15	30	8	37.5	219 ± 9
A5	15	30	6	50	201 ± 7
A6	15	30	4	75	197 ± 6

**Table 2:** Atomic composition obtained by Energy-Dispersive Spectroscopy (EDS) of the as-deposited a-Si_1−x_C_x_:H films.

Sample	RF Power (W)	CH_4_/SiH_4_	Si at. %	C at. %	O at. %
A1	6	37.5	59.28	22.81	17.92
A2	9	37.5	57.63	25.85	16.52
A3	12	37.5	52.71	32.64	14.65
A4	15	37.5	50.02	36.61	13.37
A5	15	50	49.11	39.78	11.11
A6	15	75	56.06	37.58	6.36

## Supplementary Materials

The following are available online at https://www.mdpi.com/1996-1944/13/11/2643/s1, Figure S1: PL normalized of a-Si_1−x_C_x_:H films annealed at 200 °C, (A) Ratio R_0_ = 37.5 with different RF power deposition from 6 to 15 W, (B) Ratio R_0_ from 37.5 to 75 with a fixed RF power at 15 W. Figure S2: PL normalized of a-Si_1−x_C_x_:H films annealed at 400 °C, (A) Ratio R_0_ = 37.5 with different RF power deposition from 6 to 15 W, (B) Ratio R_0_ from 37.5 to 75 with a fixed RF power at 15 W. Figure S3: PL normalized of a-Si_1−x_C_x_:H films annealed at 600 °C, (A) Ratio R_0_ = 37.5 with different RF power deposition from 6 to 15 W, (B) Ratio R_0_ from 37.5 to 75 with a fixed RF power at 15 W. Figure S4: PL normalized of a-Si_1−x_C_x_:H films annealed at 800 °C, (A) Ratio R_0_ = 37.5 with different RF power deposition from 6 to 15 W, (B) Ratio R_0_ from 37.5 to 75 with a fixed RF power at 15 W. Figure S5: PL of a-Si_1−x_C_x_:H films deposited with R_0_ = 37.5 ratio and 6 W of RF power, annealed at 200 °C, 400 °C, 600 °C, 800 °C, and as-deposited, (A) without normalization, (B) normalized. Figure S6: PL of a-Si_1−x_C_x_:H films deposited with R_0_ = 37.5 ratio and 9 W of RF power, annealed at 200 °C, 400 °C, 600 °C, 800 °C, and as-deposited, (A) without normalization, (B) normalized. Figure S7: PL of a-Si_1−x_C_x_:H films deposited with R_0_ = 37.5 ratio and 12 W of RF power, annealed at 200 °C, 400 °C, 600 °C, 800 °C, and as-deposited, (A) without normalization, (B) normalized. Figure S8: PL of a-Si_1−x_C_x_:H films deposited with R_0_ = 37.5 ratio and 15 W of RF power, annealed at 200 °C, 400 °C, 600 °C, 800 °C, and as-deposited, (A) without normalization, (B) normalized. Figure S9: PL of a-Si_1−x_C_x_:H films deposited with R_0_ = 50 ratio and 15 W of RF power, annealed at 200 °C, 400 °C, 600 °C, 800 °C, and as-deposited, (A) without normalization, (B) normalized. Figure S10: PL of a-Si_1−x_C_x_:H films deposited with R_0_ = 75 ratio and 15 W of RF power, annealed at 200 °C, 400 °C, 600 °C, 800 °C, and as-deposited, (A) without normalization, (B) normalized. Figure S11: X-ray diffraction pattern of a-Si_1−x_C_x_:H sample deposited with R_0_ = 37.5 ratio and 6 W of RF power after a fast annealing process at 800 °C.
