# Supplementary material for: Enhanced Photoluminescence of Hydrogenated Amorphous Silicon Carbide Thin Films by Means of a Fast Thermal Annealing Process
Source: Materials (Basel). 2020 Jun 10;13(11):2643. doi: 10.3390/ma13112643 (PMC7321575; doi:10.3390/ma13112643)
Supplement: Supplementary file 1 [file materials-13-02643-s001.pdf]

materials

MDPI

Supplementary Materials

Enhanced Photoluminescence of Hydrogenated Amorphous Silicon Carbide Thin Films by Means of a Fast Thermal Annealing Process

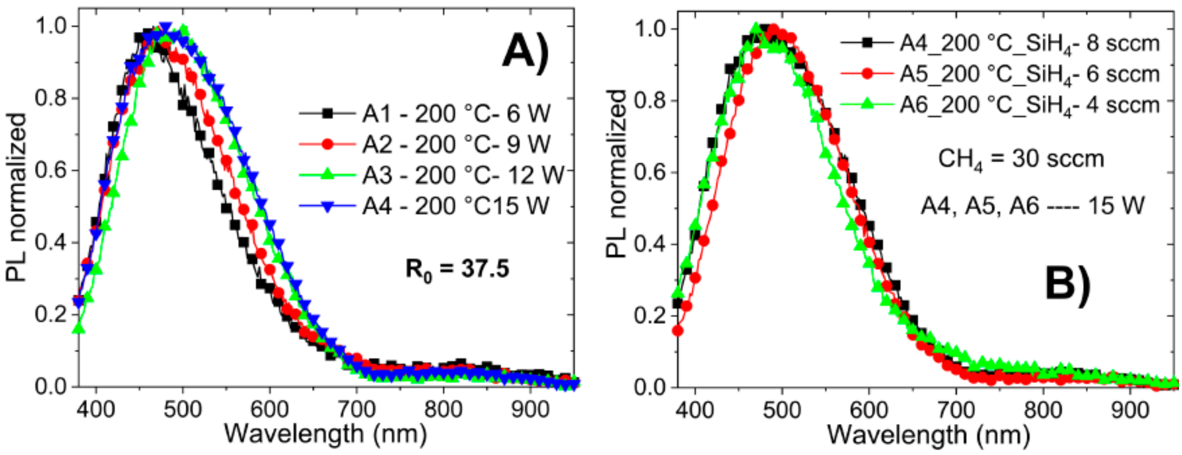

Figure S1. PL normalized of $a\text{-}Si_{1-x}C_x:H$ films annealed at 200 °C, **A)** Ratio R_0 = 37.5 with different RF power deposition from 6 to 15 W, **B)** Ratio R0 from 37.5 to 75 with a fixed RF power at 15 W.

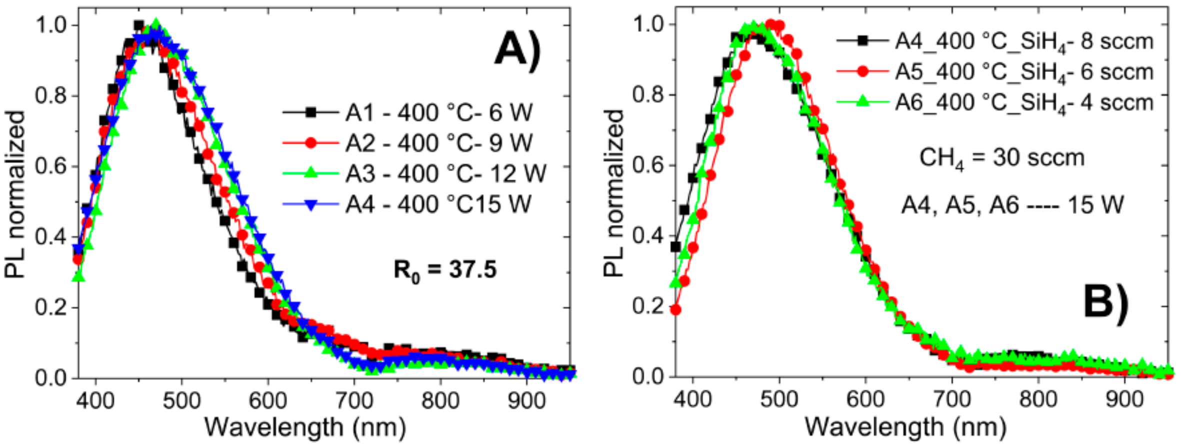

Figure S2. PL normalized of $a\text{-}Si_{1-x}C_x:H$ films annealed at 400 °C, **A)** Ratio R0 = 37.5 with different RF power deposition from 6 to 15 W, **B)** Ratio R0 from 37.5 to 75 with a fixed RF power at 15 W.

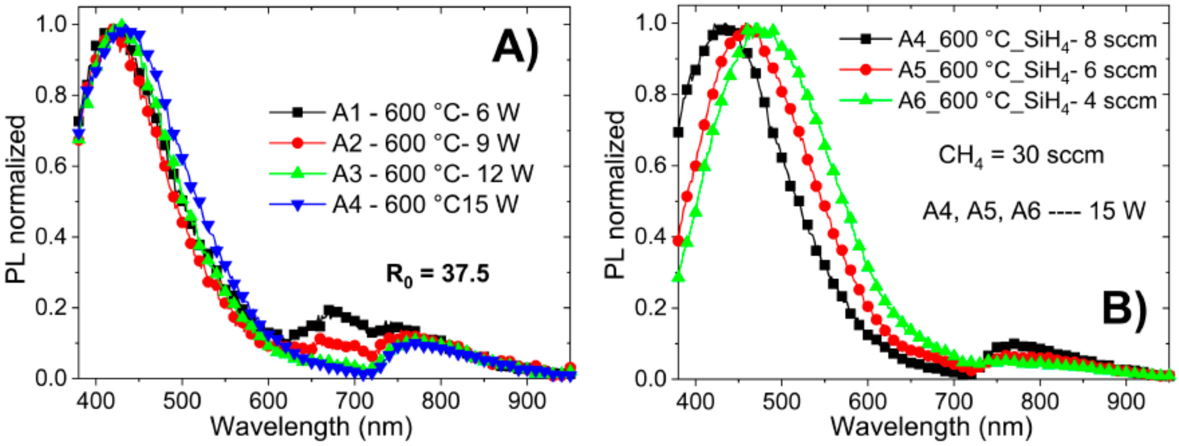

Figure S3. PL normalized of $a\text{-}Si_{1-x}C_x:H$ films annealed at 600 °C, **A)** Ratio R_0 = 37.5 with different RF power deposition from 6 to 15 W, **B)** Ratio R_0 from 37.5 to 75 with a fixed RF power at 15 W.

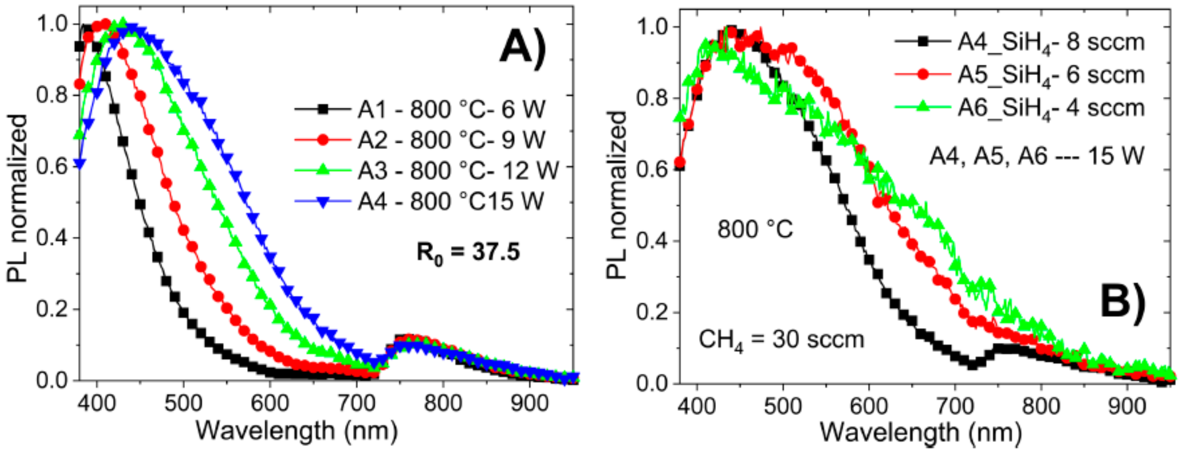

Figure S4. PL normalized of $a\text{-}Si_{1-x}C_x:H$ films annealed at 800 °C, **A)** Ratio R_0 = 37.5 with different RF power deposition from 6 to 15 W, **B)** Ratio R_0 from 37.5 to 75 with a fixed RF power at 15 W.

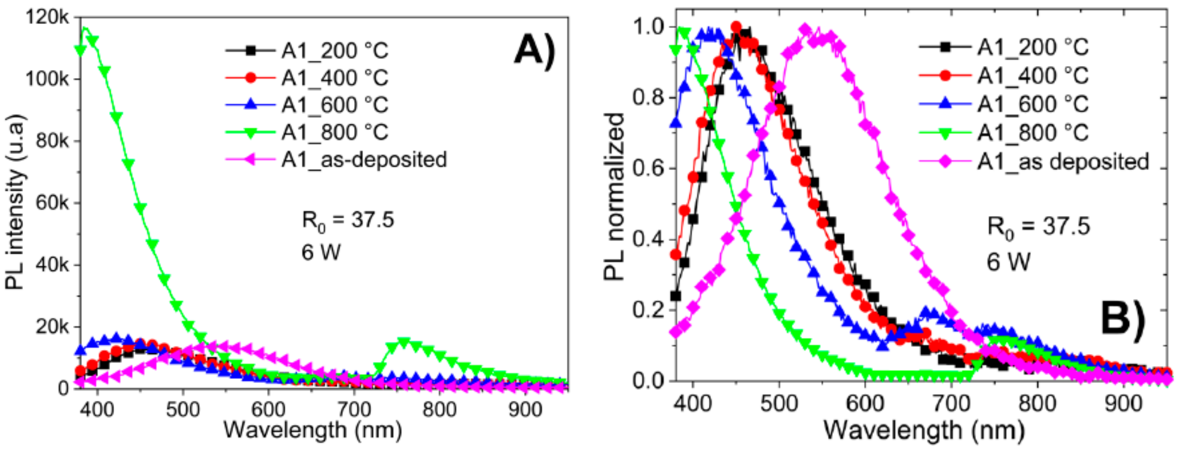

Figure S5. PL of $a\text{-}Si_{1-x}C_x:H$ films deposited with R_0 = 37.5 ratio and 6 W of RF power, annealed at 200 °C, 400 °C, 600 °C, 800 °C, and as-deposited, **A)** without normalization, **B)** normalized.

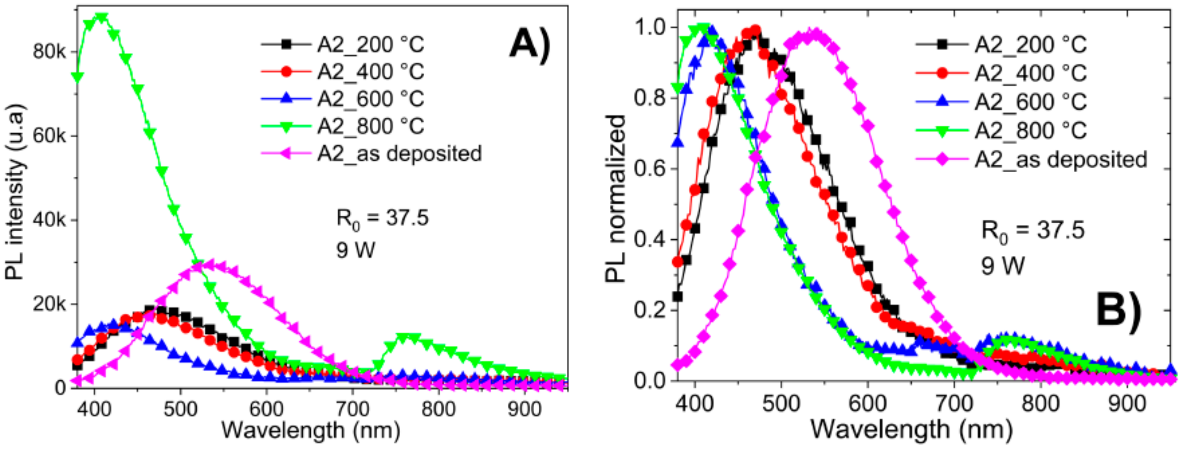

Figure S6. PL of a-$Si_{1-x}C_x$:H films deposited with R_0 = 37.5 ratio and 9 W of RF power, annealed at 200 °C, 400 °C, 600 °C, 800 °C, and as-deposited, **A)** without normalization, **B)** normalized.

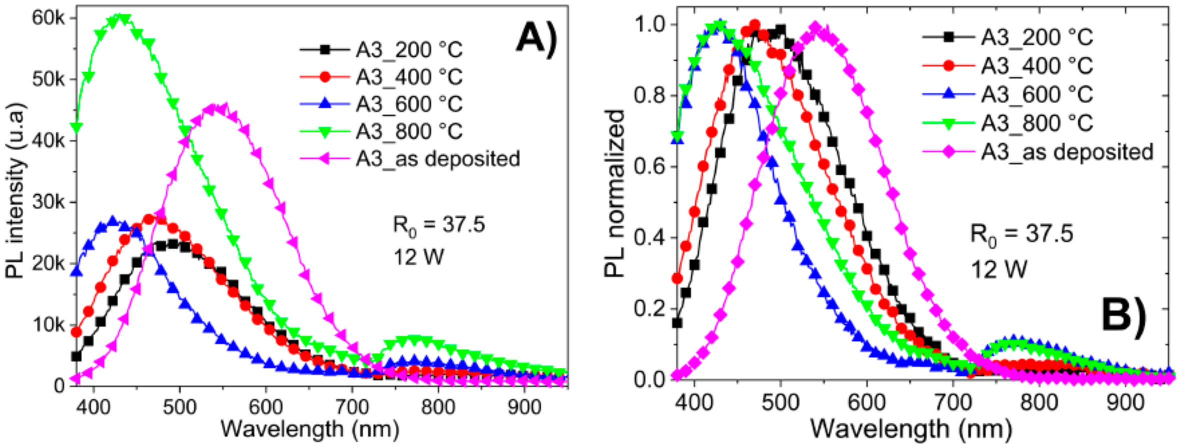

Figure S7. PL of a-$Si_{1-x}C_x$:H films deposited with R_0 = 37.5 ratio and 12 W of RF power, annealed at 200 °C, 400 °C, 600 °C, 800 °C, and as-deposited, **A)** without normalization, **B)** normalized.

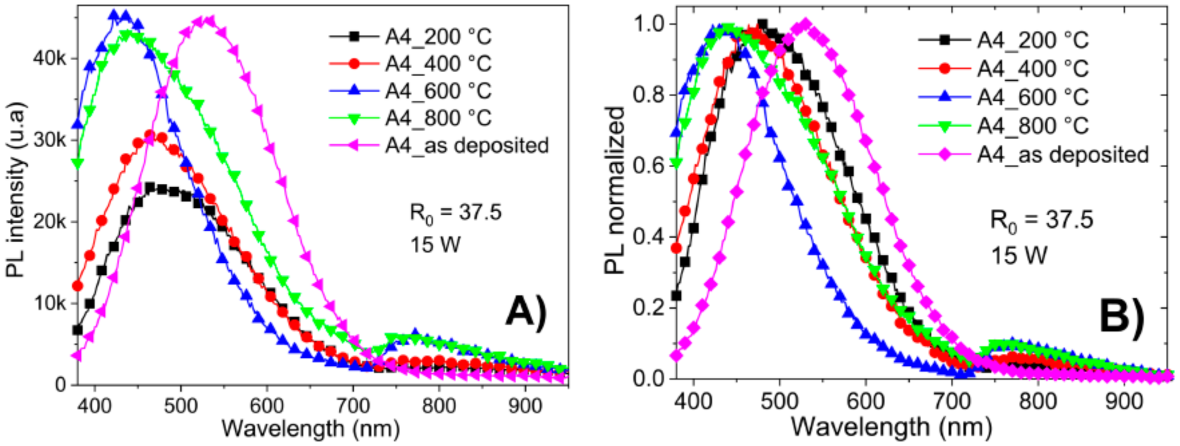

Figure S8. PL of a-$Si_{1-x}C_x$:H films deposited with R_0 = 37.5 ratio and 15 W of RF power, annealed at 200 °C, 400 °C, 600 °C, 800 °C, and as-deposited, **A)** without normalization, **B)** normalized.

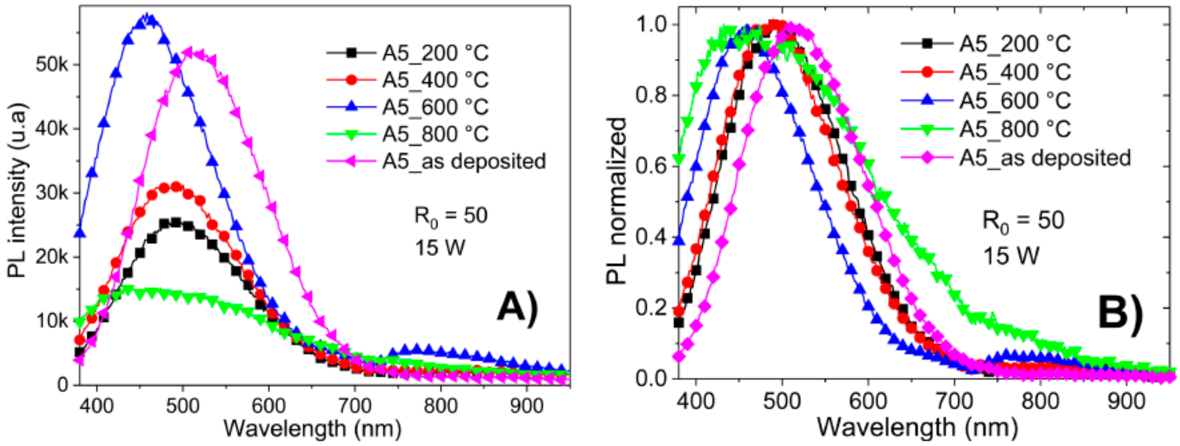

Figure S9. PL of a-$Si_{1-x}C_x$:H films deposited with R_0 = 50 ratio and 15 W of RF power, annealed at 200 °C, 400 °C, 600 °C, 800 °C, and as-deposited, **A)** without normalization, **B)** normalized.

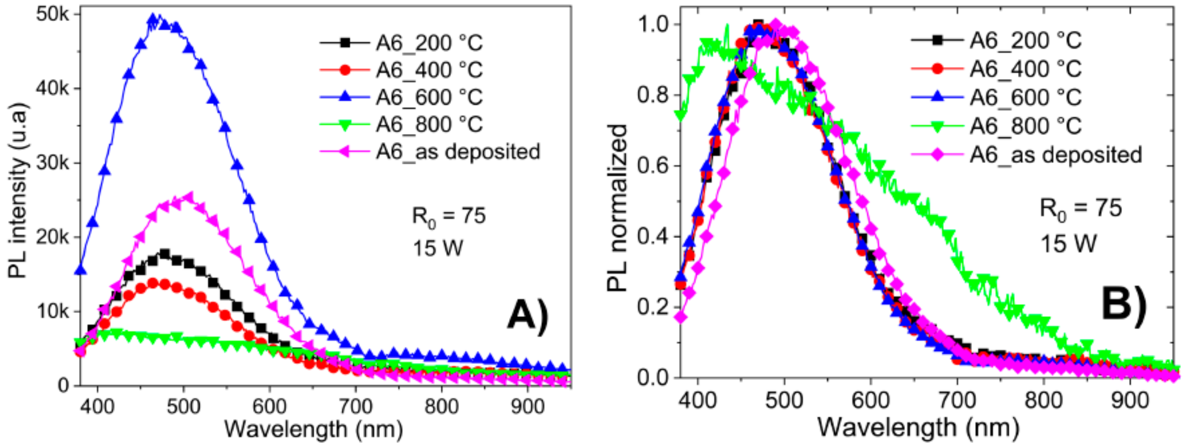

Figure S10. PL of a-$Si_{1-x}C_x$:H films deposited with R_0 = 75 ratio and 15 W of RF power, annealed at 200 °C, 400 °C, 600 °C, 800 °C, and as-deposited, **A)** without normalization, **B)** normalized.

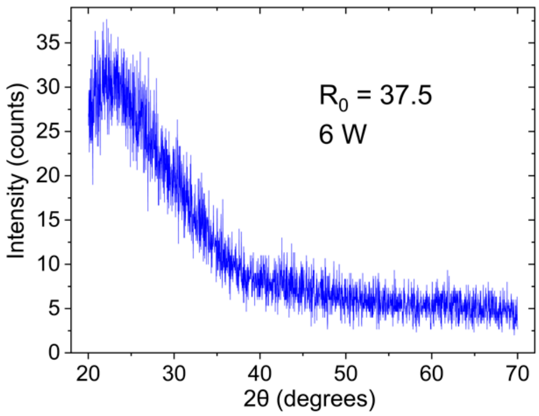

Figure S11. X-ray diffraction pattern of a-$Si_{1-x}C_x$:H sample deposited with R_0 = 37.5 ratio and 6 W of RF power after a fast annealing process at 800 °C.

© 2020 by the authors. Submitted for possible open access publication under the terms and conditions of the Creative Commons Attribution (CC BY) license (http://creativecommons.org/licenses/by/4.0/).
